# PipC affects the virulence of *Salmonella enterica* serovar *Enteritidis* and its deletion strain provides effective immune protection in mice

**DOI:** 10.3389/fmicb.2025.1631008

**Published:** 2025-06-24

**Authors:** Lu Zhang, Yubin Chen, Zhigang Yan, Yuntai Li, Xiaowen Yang, Li Chen, Yanying Zhang, Yingyu Chen, Yonghui Li, Qiumei Shi, Tonglei Wu

**Affiliations:** ^1^Hebei Provincial Key Laboratory of Preventive Veterinary Medicine, Hebei Normal University of Science and Technology, Qinhuangdao, China; ^2^Hebei Provincial Center for Livestock Breeding Improvement, Shijiazhuang, China; ^3^Key Laboratory of Animal Biosafe Risk Prevention and Control (North), Ministry of Agriculture and Rural Affairs, Institute of Animal Science, Chinese Academy of Agricultural Sciences, Beijing, China; ^4^College of Animal Medicine, Huazhong Agricultural University, Wuhan, China; ^5^The Second Hospital of Qinhuangdao, Qinhuangdao, China

**Keywords:** *Salmonella enterica* serovar *Enteritidis*, PipC, virulence, immune protection, vaccine

## Abstract

**Background:**

Salmonellosis caused by *Salmonella* sp. is a foodborne zoonotic disease that poses a significant threat to public health security. Vaccination is a safe and effective strategy for preventing and controlling *Salmonella* infections. PipC is a chaperone protein associated with *Salmonella* invasion proteins which is crucial for bacteria to invade host cells.

**Methods:**

In this study, a Δ*pipC* mutant strain was generated. Subsequently, we examined the environmental stress tolerance of the mutant strain through *in vitro* simulation experiments. Moreover, its virulence by employing cell and mouse infection models was investigated. Furthermore, we utilized a mouse model to further explore its potential as an attenuated live vaccine against *Salmonella enterica* serovar *Enteritidis* infection.

**Results:**

The *Salmonella* strain C50336 with a deletion of the *pipC* gene exhibits a significant reduction in its ability to resist environmental stress and virulence. Meanwhile, the expression levels of SPI-1-related genes (*invH*, *sipA*, *sipB*, *sipC*, *sopB*, and *sopE2*) and SPI-2-related genes (*spvB*, *ssrA*, *orf245*, *ssaS*, *ssaT*, *ssaU*, *sseB*, and *sseD*) encoding the *Salmonella* type III secretion system (T3SS) were found to be decreased, leading to a significant reduction in the bacteria’s invasion and intracellular survival abilities. The results of the mouse intraperitoneal challenge experiment showed that compared with the wild-type strain, the 50% lethal dose (LD_50_) of the Δ*pipC* strain increased by 47 times, and the bacterial loads in the liver, spleen, and cecum were significantly reduced. When mice were immunized with the Δ*pipC* mutant strain, the immunized mice showed a robust immune response, with significantly increased cytokine and antibody levels in their bodies. Mice vaccinated with the Δ*pipC* mutant strain had 100% immune protection against wild-type *Salmonella* infection.

**Conclusion:**

This study demonstrates that lack of *pipC* affects *SE* pathogenicity by decreasing its virulence both *in vitro* and *in vivo*. Vaccination of mice with Δ*pipC* conferred development of an acquired immunity and efficacious protection against experimental systemic infection. These results indicated that the Δ*pipC* mutant strain can be used in the development of attenuated live vaccines.

## Background

*Salmonella* is a facultative intracellular pathogen that belongs to the Gram-negative category and exhibits a remarkable ability to infect a diverse range of animals, including humans. This broad host range not only poses a severe threat to the healthy development of the global aquaculture industry but also undermines public health safety, resulting in substantial economic losses in various aspects (Ferrari et al., 2019). Among these, *Salmonella enteritidis* (*SE*) and *Salmonella* Typhimurium are the main serotypes that infect humans, accounting for approximately 40% of human salmonellosis cases (Cao et al., 2023). The transmission routes of *SE* to humans are diverse. It can be contracted through the consumption of contaminated food products such as pork, beef, poultry, and eggs. Additionally, in areas with poor sanitation where fecal matter exposure is more likely, the risk of human infection also increases significantly. Once infected, humans may experience a series of symptoms, including abdominal pain, diarrhea, nausea, vomiting, fever, and headaches, which can greatly affect their quality of life and overall health (Guard-Petter, 2001).

Antibiotics are commonly used to treat *Salmonella* infections, but their overuse leads to environmental pollution and accelerates the rise of multidrug-resistant strains. This not only reduces treatment effectiveness but also poses a significant threat to public health. In this context, vaccination has emerged as another crucial measure for the prevention and control of *Salmonella* infections, as emphasized by Ruvalcaba-Gómez et al. (2022) and Acevedo-Villanueva et al. (2021). Given the facultative intracellular nature of *Salmonella*, strong cellular immunity plays a vital role in clearing the pathogen. As a result, attenuated live vaccines are generally considered to offer more effective immune protection compared to other types of vaccines, as demonstrated by the research of Lin et al. (2017) and Jiang et al. (2022). Moreover, previous studies have shown that attenuated live vaccines of *Salmonella* have relatively low virulence to the host. They are capable of inducing a robust and long-lasting mucosal and humoral immune response, as pointed out by Tennant and Levine (2015). This immune response can effectively reduce bacterial adhesion and colonization within the host organism.

Currently, numerous *Salmonella* gene knockout strains have been utilized as live vaccines. For instance, Zhao et al. (2024) found in 2024 that immunizing mice with a *Salmonella* strain with the *pal* gene deleted could stimulate good immune protection. Zhang et al. (2024, 2025) found that an attenuated *Salmonella enterica* vaccine with *mcpC* and *cheV* gene knockouts was able to stimulate 100% immune protection in mice. In addition, Yin et al. (2022) and Kamble and Lee (2016) also demonstrated that attenuated vaccines prepared by deleting virulence genes such as *cpxR*, *lon*, and SPI2 were effective in reducing the colonization of wild-type strains in chickens and provided good immune protection. Overall, the exploration and improvement of *Salmonella* attenuated live vaccines based on gene deletion, are of great significance for safeguarding public health and promoting the sustainable development of the aquaculture industry. *Salmonella* Pathogenicity Island 5 (SPI-5) plays a critical role in the enteropathogenicity of *Salmonella*. It encodes five proteins, namely PipA, PipB, PipC, PipD, and SopB, that are involved in mucosal secretion and inflammatory responses in the intestine. These proteins are regulated by the type III secretion systems (T3SS) encoded by SPI-1 and SPI-2 (Wang et al., 2020). Devendra H. Shah et al. reported that *sopB* and *pipB/C* are co-regulated with SPI-1 and promote host cell invasion, suggesting that *pipC* may contribute to the invasive capacity of *Salmonella* (Darwin et al., 2001; Rodríguez-Escudero et al., 2011). Previous studies have shown that deletion of SPI-5 reduces the ability of *SE* to colonize the chicken intestine (Rychlik et al., 2009; Shah et al., 2012). Furthermore, *pipC* has been implicated in the folding of key virulence factors, including the T3SS effector protein SopB. Additionally, literature reports indicate that the expression level of *pipC* is significantly reduced in macrophages compared to bacteria in the early stationary phase (ESP) under *in vitro* conditions, suggesting a potential role of *pipC* in intracellular survival within macrophages. Taken together, these findings indicate that *pipC* may influence the virulence of *SE*, though further experimental validation is required.

To further elucidate the role of *pipC* in *Salmonella* infection and its contribution to immunoprotection, this study aims to construct a *pipC* gene deletion mutant of *SE*. The effects of this gene deletion on bacterial virulence will be evaluated through both *in vitro* and *in vivo* assays, and the immunoprotective efficacy of the deletion strain will be assessed in a mouse model.

## Materials and methods

### Bacterial strains, plasmids and cells

The bacterial strains and plasmids used in this study are shown in Table 1. *Salmonella enterica* serovar *Enteritidis* C50336 was the wild-type strain and used for constructing the Δ*pipC* mutant. The Δ*pipC* strain in this study was constructed following the *λ*-Red recombinase gene replacement method (Datsenko and Wanner, 2000). The primer sequences used for generating and confirming mutant strains are listed in Table 2. All bacterial strains were cultured on Luria-Bertani (LB) agar plates or in LB broth with necessary antibiotics at appropriate concentrations (for example, 100 μg/mL ampicillin and 34 μg/mL chloramphenicol) (Xiong et al., 2023).

**Table 1:** Strains and plasmids used in this study.

Strains or plasmids	Characteristics	Source
Strain		
C50336	*Salmonella enterica* serovar *Enteritidis*, wild-type	This study
Δ*pipC*:*cat*	A first recombination strain	
Δ*pipC*	A second recombination strain	
Δ*pipC + pipC*	Δ*pipC*-complemented strain	
Plasmids		
pKD3	Cm^R^, *cat*, FRT	The Key Laboratory of Preventive Veterinary Medicine, Hebei Province
pKD46	Amp^R^, encodes lambda red genes (exo, beta, gam), arabinose-inducible promoter for expression (ParaB)	
pCP20	Amp^R^ and Cm^R^, encode FLP recombinase	
pBR322	Amp^R^ and Tet^R^	

**Table 2:** Primers used for the construction of the *pipC* deletion mutant and complemented strain.

Primers	Sequence (5′–3′)	Product length (bp)	Purpose
P1	TTGGCAGTCAGTAAAAGGCATTTCTTCATTAATCACATCTTGAGTCTTGAGGTAACTATtgtgtaggctggagctgcttcg	1,135	Underline: *pipC* homologous fragment; Lowercase letters: *cat* homologous fragment
P2	TTGTAAAGGGCATACGTATCGCGTTTTATCTCATTAAGAAAGTATGTTGACGTATTAAAcatatgaatatcctccttag		
P3	TTATCGCCAGAGGTGCTCAATC	554 (no recombination)/1,229 (first recombination)/246 (secondary recombination)	Identification of Δ*pipC*
P4	GCCCCTTACATTTCCACCAAAG		
P5	CGGGATCCTTGGCAGTCAGTAAAAGG	501	Underline: enzyme cleavage site
P6	GCGTCGACCCACCAAAGATTCTGGTCT		
P7	TCGCTTCGCTACTTGGAG	593	Identification of the complemented strain
P8	AAGGAGCTGACTGGGTTG		

Human epithelial Caco-2 BBE cells and mouse macrophage RAW264.7 cells used in this study were provided by BeNa Culture Collection (Shanghai, China). Both cell types were cultured in DMEM (Thermo Fisher Scientific Co., Ltd.) supplemented with 10% fetal bovine serum (Thermo Fisher Scientific Co., Ltd.). Antibiotics were added as necessary, such as 50 μg/mL streptomycin and 50 U/mL penicillin, or 50 μg/mL gentamicin, in an incubator with 5% CO₂.

### Experimental animals and ethical statement

Kunming (KM) mice were obtained from Beijing Speifu Biotechnology Co., Ltd. Throughout the experiment, the mice were maintained in a sterile environment under standard housing conditions with an ambient temperature consistently kept at 22.0 ± 0.5°C and relative humidity maintained at 60 ± 10%. A 12-h light/dark cycle was established for the housing conditions. All animal experiments were conducted in full compliance with international ethical standards and the Experimental Animal Regulation Ordinances (HPDST 2020-17) stipulated by the Hebei Provincial Department of Science and Technology. The study protocol was reviewed and approved by the Animal Care and Use Committee of Hebei Normal University of Science and Technology.

### Construction of *pipC* gene deletion and complementation strains of *SE*

The *pipC* gene deletion strain in this study was constructed using the *λ* homologous recombination method (Supplementary Figure 1). Briefly, the auxiliary plasmid pKD46 was introduced into C50336 through electroporation, which encodes the Gam, Exo, and Beta proteins required for λ homologous recombination. Using pKD3 as a template, the knockout fragments were amplified by P1 and P2. The knockout fragment was then introduced into C50336 containing pKD46 via electroporation, resulting in a primary recombinant strain with chloramphenicol resistance. This strain was selected using LB agar plates containing chloramphenicol and verified using primers P3 and P4. The positive strain obtained was named Δ*pipC*:*cat*. The pCP20 plasmid, which encodes the Flp recombinase, was able to excise the *cat* gene from the knockout fragment. The pCP20 plasmid was introduced into Δ*pipC*:*cat* by electroporation, resulting in a secondary recombinant strain. This strain was verified using primers P3 and P4. The positive strain obtained was named Δ*pipC*.

To construct the complement strain, the nucleic acids of C50336 were used as a template, and the complement fragment was amplified using P5 and P6. The complement fragment and pBR322 vector plasmid were digested with restriction endonucleases *BamH I* and *Sal I*, respectively. The two digested fragments were ligated using T4 DNA ligase. The recombinant vector was introduced into Δ*pipC* by electroporation and verified with P7 and P8. The positive strain was named Δ*pipC* + *pipC*.

### Genetic stability testing

To determine the genetic stability of the Δ*pipC*, it was serially passaged 40 times in LB medium, every 12 h. Nucleic acids from liquid cultures are extracted every other generation and PCR verification using P3 and P4.

### Growth characteristics assay

The overnight cultures of C50336, Δ*pipC*, and Δ*pipC* + *pipC* were subcultured at a 1:100 ratio into 5 mL of LB liquid medium and incubated at 37°C in a shaking incubator. Growth was determined by monitoring the absorbance of bacterial cultures at 600 nm (OD_600_ values). Growth curves were plotted based on the growth of each strain at different time points.

### *In vitro* stress simulation experiments

Overnight cultures of C50336, Δ*pipC*, and Δ*pipC* + *pipC* were washed three times with PBS and resuspended in the original volume. The bacterial counts before stress were determined using the traditional plate count method. The bacterial suspensions were exposed to acid stress (pH 3.5), alkaline stress (pH 10.0), and heat stress (42°C) for 1 h, as well as to oxidative stress (10 mmol/L H_2_O_2_) for 30 min. After stress exposure, the bacterial counts were determined. The survival rate of each strain under different conditions was calculated as follows: survival rate = (post-stress bacterial count)/(initial bacterial count).

### Cell culture

The human epithelial cancer cell lines Caco-2 were cultured in Dulbecco’s modified Eagle medium (DMEM) supplemented with 20% fetal bovine serum (FBS) and 1% penicillin-streptomycin solution. The mouse macrophage RAW264.7 was cultured in DMEM containing 10% FBS and 1% penicillin-streptomycin solution. When the cells reached 80% confluence, the monolayers were washed three times with PBS. The cells were then seeded in 12-well tissue culture plates at a density of 1 × 10^6^ cells/well. The plates were incubated at 37°C in an atmosphere containing 5% CO_2_.

### Adherence and invasion assays

To investigate the impact of *pipC* gene deletion on the adhesion and invasion ability of *SE*, the overnight cultures of C50336, Δ*pipC* and Δ*pipC* + *pipC* were washed three times with PBS and subsequently resuspended. The number of bacteria per mL of bacterial suspension (number of infected bacteria) was measured. Following the washing of confluent cell monolayers with DMEM, C50336, Δ*pipC* and Δ*pipC* + *pipC* were inoculated, respectively, onto the Caco-2 cells at a multiplicity of infection (MOI) of 100:1 and were incubated for 1 h at 37°C under 5% CO_2_ (Xiong et al., 2023).

### Adhesion assay

For bacterial adhesion, the cells were washed, and then incubated with PBS containing Triton X-100 (0.5%) at 37°C for 10 min. The cell lysates were serially diluted and inoculated onto LB agar for counting. The number of bacteria per mL of cell lysate (number of adherent bacteria) was measured. The adhesion rate was calculated using the formula: Adhesion rate = (number of adherent bacteria/number of infected bacteria) × 100%.

### Invasion assay

For bacterial invasion, 1 h after bacterial colonization, the cells were incubated for an additional 1 h in DMEM with gentamicin (100 μg/mL), washed and incubated with PBS containing Triton X-100 (0.5%) at 37°C for 10 min. The cell lysates were serially diluted and inoculated onto LB agar for counting. The number of bacteria per mL of cell lysate (number of invading bacteria) was measured. Invasion rate = (number of invading bacteria/number of infected bacteria) × 100%.

### Intracellular proliferation assay

To evaluate the survival rate of Δ*pipC* in phagocytic cells, after infecting the cells with bacteria for 2 h as described above, DMEM containing 100 μg/mL gentamicin was added and incubated for 1 h. One group of cells was then washed and lysed with 0.5% Triton X-100, and the intracellular bacteria were counted (intracellular bacteria at 3 h). Another group of cells was washed and incubated with DMEM containing 10 μg/mL gentamicin at 37°C for 20 h. These cells were also lysed with 0.5% Triton X-100, and the intracellular bacteria were counted (intracellular bacteria at 23 h). Intracellular survival rate = (intracellular bacteria at 23 h/intracellular bacteria at 3 h) × 100%.

### Assessment of bacterial virulence

A total of 75 six-week-old KM mice were randomly divided into 15 groups (*n* = 5). These groups were categorized into three sets: five groups for the Δ*pipC*, five groups for the C50336, and the remaining five groups designated as the Δ*pipC* + *pipC* group. Mice in the Δ*pipC* groups were intraperitoneally (i.p.) inoculated with Δ*pipC* containing of 1.68 × 10^9^, 1.68 × 10^8^, 1.68 × 10^7^, 1.68 × 10^6^ or 1.68 × 10^5^ CFU/mouse, respectively. Similarly, the C50336 groups were i.p. inoculated with C50336 containing of 2 × 10^7^, 2 × 10^6^, 2 × 10^5^, 2 × 10^4^ or 2 × 10^3^ CFU/mouse, respectivly. The Δ*pipC* + *pipC* groups were i.p. inoculated with Δ*pipC* + *pipC* containing of 2 × 10^7^, 2 × 10^6^, 2 × 10^5^, 2 × 10^4^ or 2 × 10^3^ CFU/mouse, respectively. Five additional mice were i.p. injected with the same volume of PBS as a negative control.

The number of dead mice was recorded for 14 days and LD_50_ was calculated, which was calculated using the formula of log_10_ [50% endpoint] = A + (B × C), where A = log_10_ [infectious dose showing a mortality next below 50%], B = difference of logarithms = [50% − (mortality at infectious dose next below 50%)]/[(mortality next above 50%) − (mortality next below 50%)], and C = log_10_ [difference between serial infectious doses used in challenge studies] (Park et al., 2022; Zhang et al., 2019).

### RNA extraction and qPCR

In order to further investigate the effect of *pipC* gene deletion on the virulence of *SE*, qPCR was used to measure the expression levels of virulence genes in C50336, Δ*pipC*, and Δ*pipC* + *pipC*. In short, the bacteria were cultured to the logarithmic phase and total RNA was extracted using an RNA extraction kit (Aidlab, Beijing, China). Complementary DNA (cDNA) was synthesized using a reverse transcription kit (TOYOBO, Osaka, Japan). Primers were designed by referring to previous literature (Upadhyaya et al., 2013). The primer sequences used for qPCR are listed in Table 3. Using cDNA as a template, the relative gene expression was quantified using the comparative critical threshold (Ct) method through qPCR. Data were normalized to the endogenous control (*16S rRNA*), and the level of candidate gene expression between treated and control samples were determined. The qPCR thermal cycling conditions were as follows: initial denaturation at 95°C for 10 min, followed by 40 cycles of 95°C for 15 s and 60°C for 1 min.

**Table 3:** The qPCR primers of virulence genes.

Primers	Sequence (5′–3′)
*16S-*F	CCAGGGCTACACACGTGCTA
*16S-*R	TCTCGCGAGGTCGCTTCT
*invH-*F	CCCTTCCTCCGTGAGCAAA
*invH-*R	TGGCCAGTTGCTCTTTCTGA
*orf245-*F	CAGGGTAATATCGATGTGGACTACA
*orf245-*R	GCGGTATGTGGAAAACGAGTTT
*sipA-*F	CAGGGAACGGTGTGGAGGTA
*sipA-*R	AGACGTTTTTGGGTGTGATACGT
*sipB-*F	GCCACTGCTGAATCTGATCCA
*sipB-*R	CGAGGCGCTTGCTGATTT
*ssrA-*F	CGAGTATGGCTGGATCAAAACA
*ssrA-*R	TGTACGTATTTTTTGCGGGATGT
*spvB-*F	TGGGTGGGCAACAGCAA
*spvB-*R	GCAGGATGCCGTTACTGTCA
*ssaS*-F	CGCAACTTTTATGGATCGTC
*ssaS*-R	TGTAGCGTTTGGTCCTGTATT
*sipC*-F	TCTGGCAAATAATGTCACGA
*sipC*-R	CGCTCTGGGAAATACTACCG
*sopB*-F	ACCCGCCTGGAATTGTAA
*sopB*-R	GAAAGATTGAGCACCTCTGG
*ssaT*-F	TGCTTATGTCACTTACCTTTCC
*ssaT*-R	AATATCGTACCCATTGTCGC
*ssaU*-F	TATTGCGGTTTGTCTTGGC
*ssaU*-R	GGGATGCAGTTGCGTTCA
*sseB*-F	CTTATCCCAGCAAAATCCG
*sseB*-R	TTAGCAATCACCTCATCCATCT
*sseD*-F	CTTCTTCCACTCCATCTCCC
*sseD*-R	CGTCTGTAAAACATTGACTTGC
*sopE2*-F	TAACACTATCCACCCAGCACT
*sopE2*-R	TTAATACCGCCCTACCCTC

### Bacterial colonization assay

Thirty mice were randomly divided into two groups (*n* = 15). One group was intraperitoneally injected with 1 × 10^4^ CFU/mouse of Δ*pipC*, while the other group received an equal dose of C50336. Additionally, three mice were injected with 200 μL of PBS as a negative control. At predetermined time points (3 days, 7 days, and 14 days), five mice from each group were randomly selected and euthanized. Their spleens, livers, and cecums were collected, homogenized in PBS, and subsequently serially diluted before being evenly spread on XLT4 agar. The number of CFUs for each sample was determined 12 h later. For this animal study, all mice were anesthetized using a 20% urethane (ethyl carbamate) solution, and every effort was made to ensure humane treatment of the animals.

### Vaccination schedules of oral attenuated live vaccine and sample collection

Twenty-four female KM mice, aged 8 weeks, were randomly divided into two groups (*n* = 12): an immunization group and a control group. On day 0, the immunization group received an oral administration of 1 × 10^6^ CFU of Δ*pipC*, whereas the control group was orally administered 200 μL of PBS. A booster immunization with an identical dose was given to the immunization group on day 14 dpi, while the control group received an equivalent volume of PBS.

**Figure 1:**
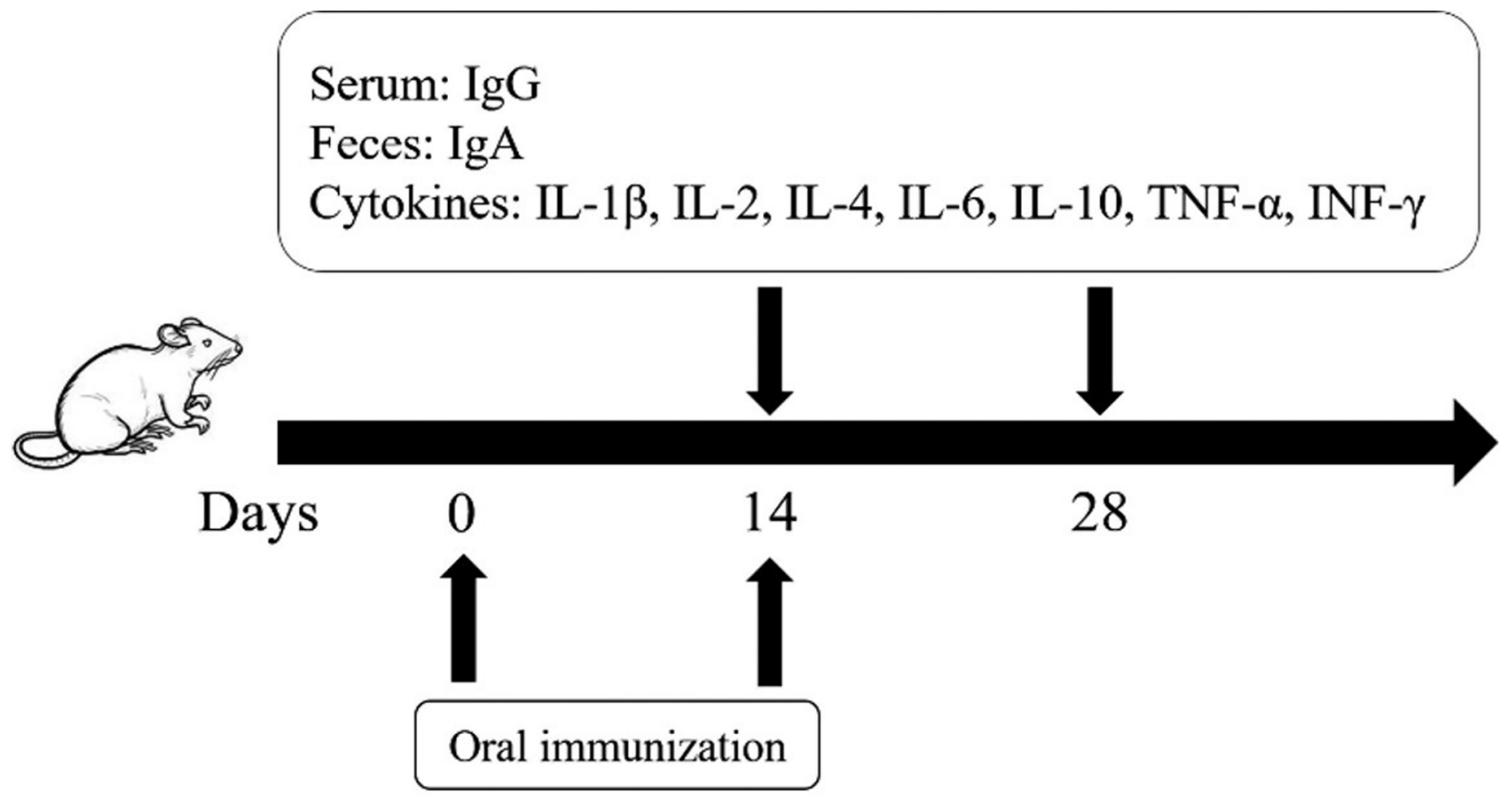
The vaccination scheme and detection protocol designed in this study.

At 14 dpi and 28 dpi, 6 mice from each group were randomly euthanized, and spleens were collected for splenic lymphocyte stimulation test and detection of cytokine expression levels. Additionally, serum and feces were collected from 3 mice for IgG and SIgA detection. The vaccination regimen of this study is shown in Figure 1.

### *Salmonella* soluble antigen preparation

C50336 was cultivated overnight at 37°C, 180 rpm with shaking until it reached the logarithmic phase. The bacteria were centrifuged at a speed of 13,000 × g at 4°C, washed three times with PBS, and resuspended in PBS to a concentration of 1 × 10^10^ CFU/mL. Use SCIENTZ ultrasonic homogenizer (SCIENTZ-IID, Ningbo, China) to sonicate the cells and centrifuge the 13,000 × g sample at 4°C to granulate the fragments. The sample was sterilized through a 0.22 μm PES (polyethersulfone) filter (Merck, Darmstadt, Germany) (Ji et al., 2022).

### Splenic lymphocytes stimulation test

Lymphocytes were isolated from each group of three spleen samples at 14 and 28 dpi. After Trypan blue dye exclusion testing, suspensions of splenic mononuclear cells (1 × 10^7^ cells/well) were cultured in Roswell Park Memorial Institute 1,640 medium (RPMI-1640) supplemented with 10% FBS and 100 μg/mL penicillin-streptomycin within 96-well tissue culture plates. These cultures were then incubated with 10 μg/mL soluble antigen or without any stimulant as a negative control, under conditions of 37°C and 5% CO₂ for a duration of 72 h. Lymphocyte proliferation was measured using an MTT kit (Beyotime, Shanghai, China). The cell proliferation was expressed as the stimulation index (SI), which was calculated using the equation: SI = (OD_570_ of the antigen-stimulated cells)/(OD_570_ of the unstimulated cells) (Kang et al., 2021).

### The expression of cytokines in the spleen

Quantitative real-time PCR (qPCR) was employed to assess the mRNA expression levels of splenic cytokines, including IL-1β, IL-2, IL-4, IL-6, IL-10, TNF-α, and IFN-γ, at 14 and 28 days post-immunization (dpi). Total RNA was extracted from spleen tissues using Triquick Reagent (Solarbio, Beijing, China), and first-strand cDNA was synthesized with a cDNA synthesis kit (TOYOBO, Osaka, Japan). Samples were stored at −80°C until further use. qPCR was performed using the Ultra SYBR Green Mixture (CWBio, Jiangsu, China) on a Lepgen-96 Real-Time PCR System (LEpu, Lepgen-96, China). The primer sequences used for qPCR are listed in Table 4. Cytokine expression levels were normalized to the internal reference genes gapdh and β-actin, and relative expression was calculated using the 2^−ΔΔCt^ method (Kang et al., 2021). The thermal cycling conditions were as follows: initial denaturation at 95°C for 10 min, followed by 40 cycles of denaturation at 95°C for 15 s and annealing/extension at 60°C for 1 min.

**Table 4:** The qPCR primers of cytokines.

Primers	Sequence (5′–3′)
gapdh-F	AGGTCGGTGTGAACGGATTTG
gapdh-R	TGTAGACCATGTAGTTGAGGTCA
β-actin-F	TTCAACACCCCAGCCATG
β-actin-R	CCTCGTAGATGGGCACAGT
IL-1β-F	GACTGTTTCTAATGCCTTCCC
IL-1β-R	ATGGTTTCTTGTGACCCTGA
IL-2-F	TGAGCAGGATGGAGAATTACAGG
IL-2-R	GTCCAAGTTCATCTTCTAGGCAC
IL-4-F	GGTCTCAACCCCCAGCTAGT
IL-4-R	GCCGATGATCTCTCTCAAGTGAT
IL-6-F	TAGTCCTTCCTACCCCAATTTCC
IL-6-R	TTGGTCCTTAGCCACTCCTTC
IL-10-F	CTTACTGACTGGCATGAGGATCA
IL-10-R	GCAGCTCTAGGAGCATGTGG
IFN-γ-F	ATGAACGCTACACACTGCATC
IFN-γ-R	CCATCCTTTTGCCAGTTCCTC
TNF-α-F	CCCTCACACTCAGATCATCTTCT
TNF-α-R	GCTACGACGTGGGCTACAG

### Detection of IgG and SIgA

To examine the antibody responses, serum and feces samples were collected from 3 mice per group at 14 and 28 dpi after inoculation with the initial and booster doses of Δ*pipC*. Enzyme-linked immunosorbent assay (ELISA) was employed to quantify the serum IgG and fecal IgA (SIgA) responses against the soluble antigen derived from *SE*. For serum extraction, whole blood samples were centrifuged for 10 min at 10,000 × g at 4°C. The resultant supernatant, containing the serum, was carefully separated from the pellet and preserved at −20°C until use. To obtain the fecal supernatant, the feces samples were weighed, followed by the addition of a 25% weight/volume (w/v) solution of fecal slurry comprised of 0.01% sodium azide, 1% protease inhibitor in PBS. The samples were homogenized by vortexing for 15 min. After centrifugation, collect the supernatant and store it at −80° C for IgA detection.

The ELISA plates were coated with *Salmonella* soluble antigen (1 μg/well) and incubated overnight at 4°C. Wells were blocked with 5% skim milk at 37°C for 2 h (200 μL/well), followed by washing three times with PBS containing 0.05% Tween-20 (PBST) (280 μL/well). Dilute the serum sample at 1:200 and add it to the well, then incubate at 4°C for 1 h (100 μL/well), and wash three times with PBST. The secondary Ab goat anti-mouse IgG-HRP diluted 1:10,000 was added (100 μL/well) (Applygen, Beijing, China). The Ab was allowed to interact with the samples for 35 min at 37°C, followed by washing three times with PBST. Add 3,3′,5,5′-tetramethylbenzidine (TMB) substrate (100 μL/well) and incubate at 37°C for 10 min. The reaction was terminated by adding 50 μL of 2 M H_2_SO_4_ to each well, and the absorbance was read at 450 nm in plate reader (Tecan, Shanghai, China).

For IgA, the ELISA plates were coated with 1 μg *Salmonella* soluble antigen per well and incubated overnight. Wells were blocked with 5% skim milk at 37°C for 2 h, followed by washing three times with PBST. The undiluted fecal supernatant was added to the well (100 μL/well), followed by incubated at 4°C for 1 h. The secondary Ab goat anti-mouse IgA-HRP diluted 1:10,000 was added, and the rest of the protocol was performed as described above (Emerson et al., 2022).

### Immune protection assessment for the Δ*pipC*

To evaluate the immune protection of Δ*pipC*. Twenty 6-week-old female KM mice were randomly divided into 2 groups (*n* = 10), namely the vaccinated group and the unvaccinated group. The vaccinated group was orally immunized with 1 × 10^6^ CFU/mouse of Δ*pipC*, while the unvaccinated group was orally immunized with 200 μL of PBS. In addition, another 10 mice without vaccination and challenge were used as the control group. At 14 dpi, the vaccinated mice received the same dose of Δ*pipC* for enhanced immunity. At 28 dpi, the vaccinated group and unvaccinated group were challenged with 2 × 10^7^ CFU/mouse of C50336 by intraperitoneal injections. Deaths and clinical symptoms were recorded daily for 14 d post the challenge (dpc), and calculate the relative survival rate according to the formula: Relative survival rate = (mortality rate of the unvaccinated group − mortality rate of the vaccinated group)/mortality rate of the unvaccinated group × 100%.

### Statistical analysis

Statistical analysis was conducted using GraphPad Prism 9 (GraphPad Software, CA, United States) and IBM SPSS (IBM Corporation, Armonk, NY, United States) software. Data are expressed as the mean ± standard error of the mean (SEM). All statistical analysis were two-way ANOVA and post-test. Differences between two samples were evaluated using Student’s *t*-test. Significant differences are indicated with an asterisk (*), where *: *p* < 0.05, **: 0.001 < *p* < 0.01, and ***: *p* < 0.001 are considered to represent statistically significant differences in mean values. ns means not significant (Kirthika et al., 2020).

## Results

### The *ΔpipC* and complementation strain were successfully constructed

**Figure 2:**
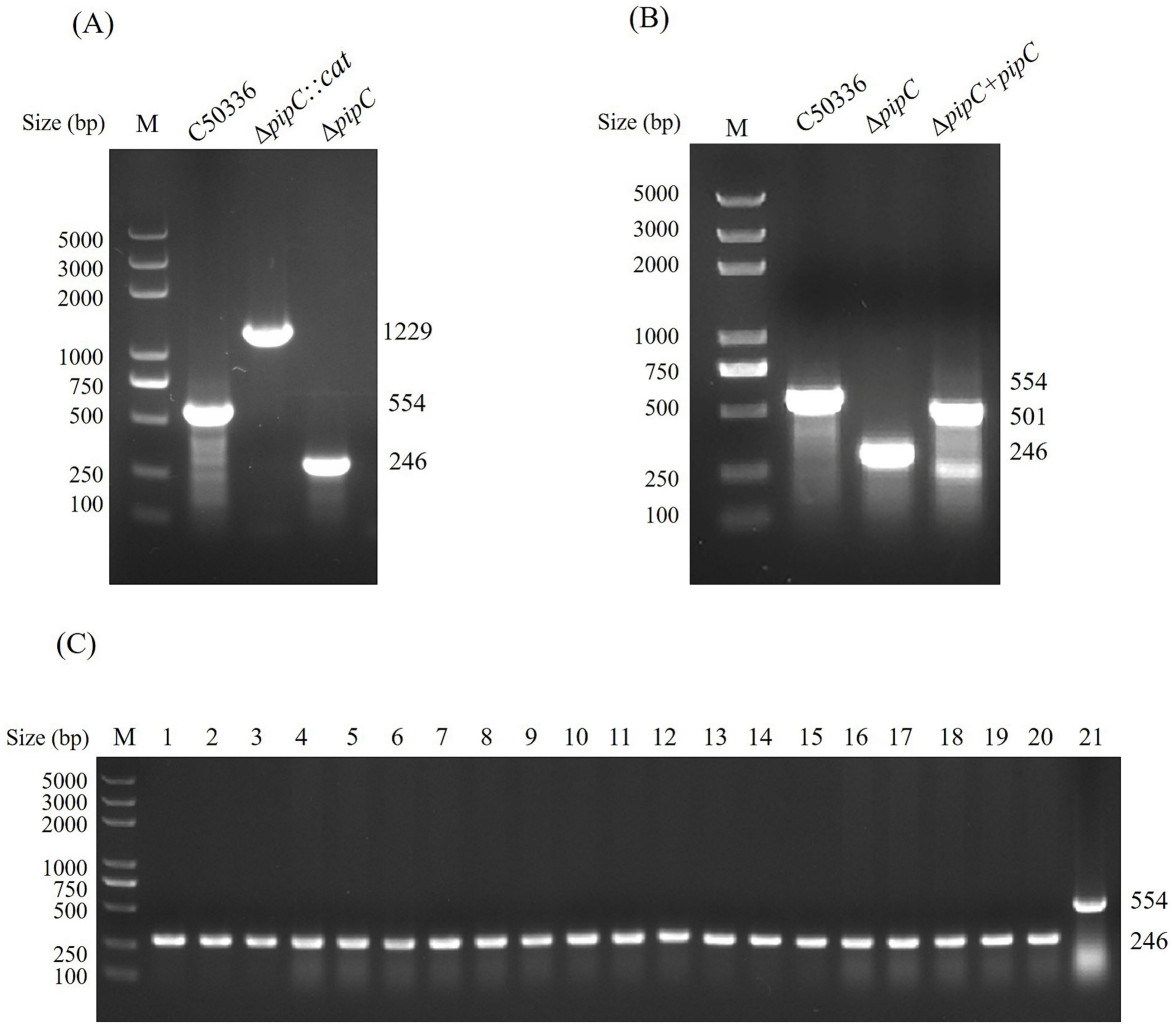
**(A)** PCR identification of Δ*pipC* and Δ*pipC*:*cat* mutants. The wild-type strain C50336 yields a 554 bp amplicon corresponding to the intact *pipC* gene. In contrast, the Δ*pipC* mutant produces a 246 bp fragment, while the Δ*pipC*:*cat* strain generates a 1,229 bp product. **(B)** PCR confirmation of the Δ*pipC* complemented strain. The Δ*pipC* + *pipC* strain yields a 594 bp PCR product, indicating successful complementation. **(C)** Assessment of Δ*pipC* genetic stability. Lanes 1–20 show 246 bp PCR products from sequential passages of the Δ*pipC* mutant, and lane 21 displays a 554 bp band from the wild-type C50336 strain.

We constructed a *pipC* gene deletion strain of *SE* by using the *λ*-Red homologous recombination method and constructed a complementation strain by using the pBR322 plasmid. The primers P3 and P4, P7 and P8 were used to identify Δ*pipC* and Δ *pipC* + *pipC*, respectively. As shown in Figures 2A,B, the *pipC* gene deletion strain and complementation strain have been successfully constructed.

### Δ*pipC* has good genetic stability

The Δ*pipC* mutant was serially passaged 40 times in LB medium, and the presence of the *pipC* deletion was then assessed by PCR (Figure 2C). The *pipC* deletion was still detectable in the Δ*pipC* mutant strain, indicating that this strain has good genetic stability.

### The *pipC* gene does not affect the growth ability of *SE*

**Figure 3:**
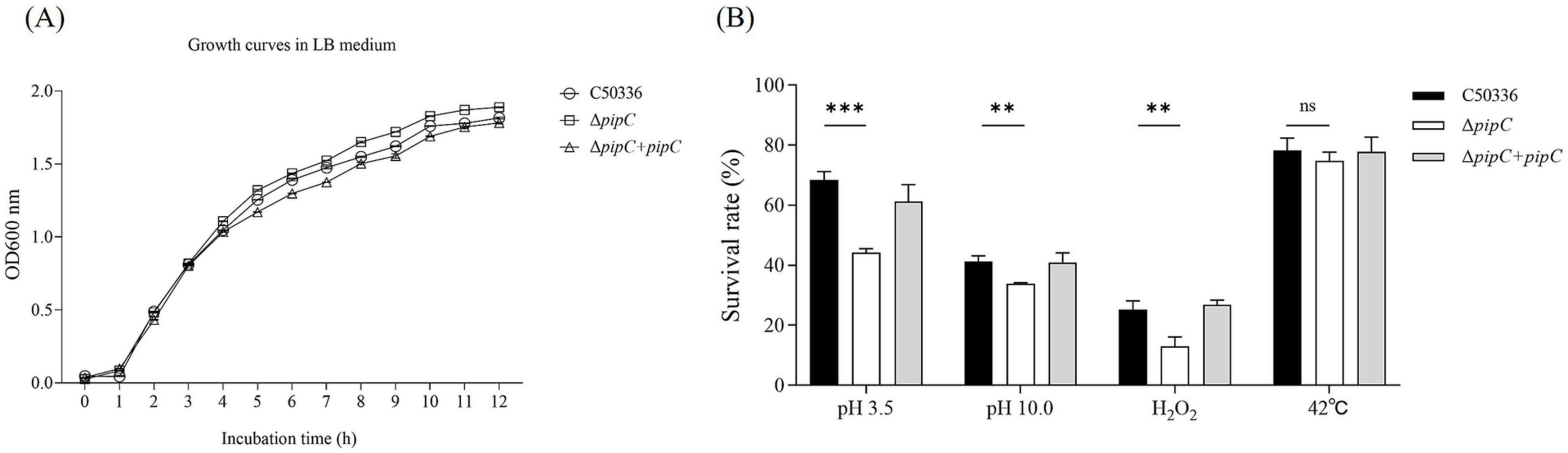
**(A)** Growth curves for the C50336, Δ*pipC* and Δ*pipC* + *pipC*. All strains were cultured in LB medium. **(B)** Survival rate of Δ*pipC* under different environmental stress. Data are presented as the mean ± SD of three independent replicates. Statistical significance was determined as *p* < 0.01 (**) and *p* < 0.001 (***).

As shown in Figure 3A, the growth characteristics of C50336, Δ*pipC* and Δ*pipC* + *pipC* in LB medium did not differ greatly. This indicated that *pipC* deletion did not influence the growth characteristics of *SE*.

### The deletion of *pipC* gene weakens the resistance of *SE* to environmental stress

To investigate whether the deletion of the *pipC* gene affects the resistance of *SE* to environmental stress, the survival rates of C50336, Δ*pipC*, and Δ*pipC* + *pipC* were compared under acid stress (pH 3.5), alkaline stress (pH 10.0), oxidative stress (H_2_O_2_), and heat stress (42°C). The results shown in Figure 3B reveal that, compared to C50336, the survival rate of Δ*pipC* was significantly reduced under acidic, alkaline, and oxidative stress conditions, whereas no statistically significant difference was observed under heat stress conditions. Since *pipC* deletion did not affect the growth characteristics of *SE*, the reduced survival rate of the mutant strain can be attributed to the role of the *pipC* gene in enhancing resistance to acid, alkaline, and oxidative stress.

### The Δ*pipC* mutant shows attenuated virulence *in vitro*

**Figure 4:**
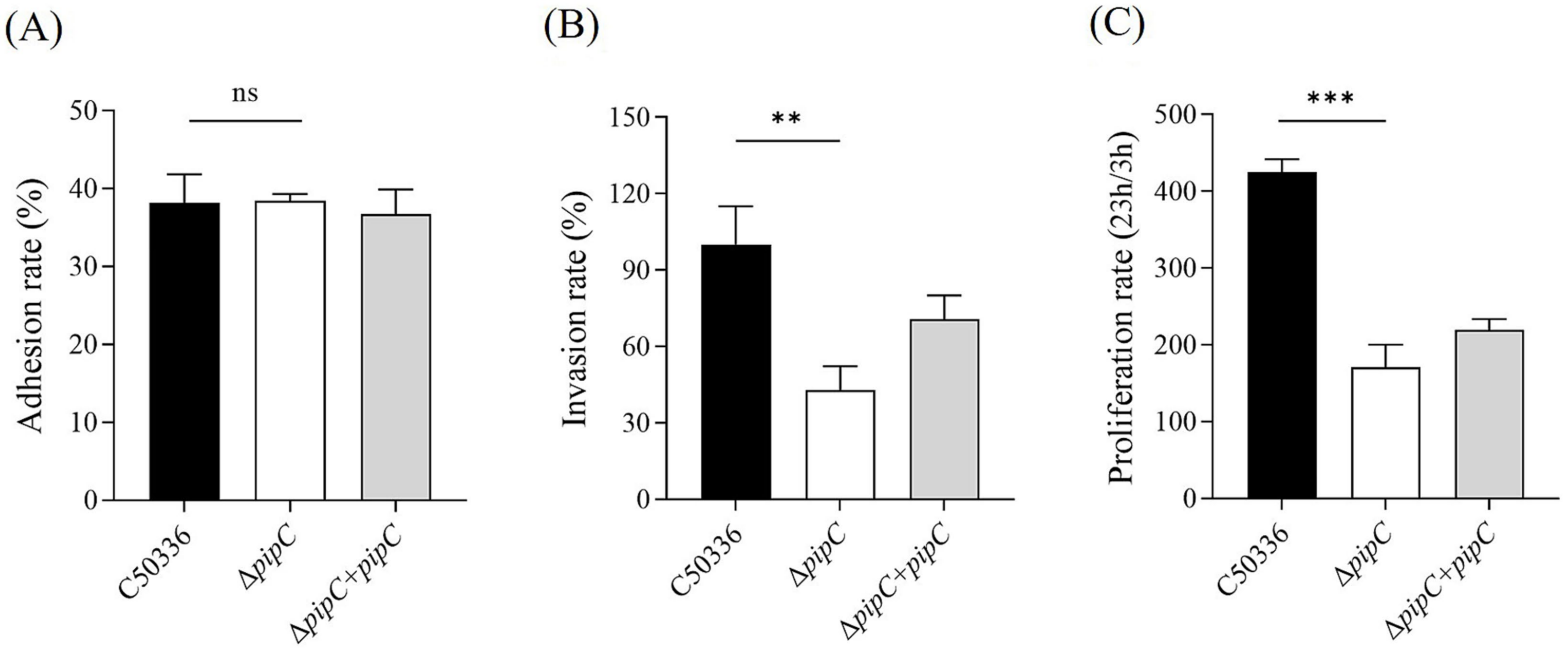
**(A)** Adherence and **(B)** invasion assays for C50336, Δ*pipC* and Δ*pipC* + *pipC* to Caco-2. The invasion rate of C50336 is considered to be 100%, and the invasiveness of Δ*pipC* or Δ*pipC* + *pipC* is calculated as its percentage. **(C)** Intracellular survival assay for C50336, Δ*pipC* and Δ*pipC* + *pipC* to RAW264.7. Data are presented as the mean ± SD of three independent replicates. Statistical significance was determined as *p* < 0.05 (*) and *p* < 0.01 (**).

The adhesion rate and invasion rate in human epithelial Caco-2, and the intracellular survival rate in mouse macrophage RAW264.7 of Δ*pipC* and C50336 was determined. As shown in Figure 4A, the adhesion rate of bacteria was not different between C50336 and Δ*pipC* in Caco-2. However, the invasion rate in Caco-2 and the intracellular survival rate in RAW264.7 of Δ*pipC*, compared to of C50336, showed significantly reduced (Figures 4B,C). These results indicate that *SE* with *pipC* deletion shows attenuated virulence *in vitro*.

### The Δ*pipC* exhibits reduced virulence in a mouse model

The virulence of the Δ*pipC* and C50336 strains was evaluated in 6-week-old KM mouse after i.p. challenge. As shown in Table 5, the LD_50_ of Δ*pipC* was 2.99 × 10^7^ CFU, which was 47-fold higher than that of the wild-type C50336 (2.99 × 10^7^/6.32 × 10^5^ ≈ 47). The LD_50_ of Δ*pipC*+*pipC* is 1 × 10^6^ CFU/mouse. The result indicated that the virulence of the Δ*pipC* was attenuated compared to the C50336.

**Table 5:** The LD_50_ of the C50336, Δ*pipC* and Δ*pipC + pipC* in mice.

Group	Dose (CFU/mouse)	Number of deaths/Total number of mice	LD_50_ (CFU)
C50336	2 × 10^7^	5/5	6.32 × 10^5^
	2 × 10^6^	3/5	
	2 × 10^5^	2/5	
	2 × 10^4^	0/5	
	2 × 10^3^	0/5	
Δ*pipC*	1.68 × 10^9^	5/5	2.99 × 10^7^
	1.68 × 10^8^	4/5	
	1.68 × 10^7^	2/5	
	1.68 × 10^6^	0/5	
	1.68 × 10^5^	0/5	
Δ*pipC + pipC*	2 × 10^7^	5/5	1 × 10^6^
	2 × 10^6^	3/5	
	2 × 10^5^	1/5	
	2 × 10^4^	0/5	
	2 × 10^3^	0/5	
Blank	PBS	0/5	/

### The deletion of *pipC* results in the downregulation of multiple virulence gene expression levels in *SE*

**Figure 5:**
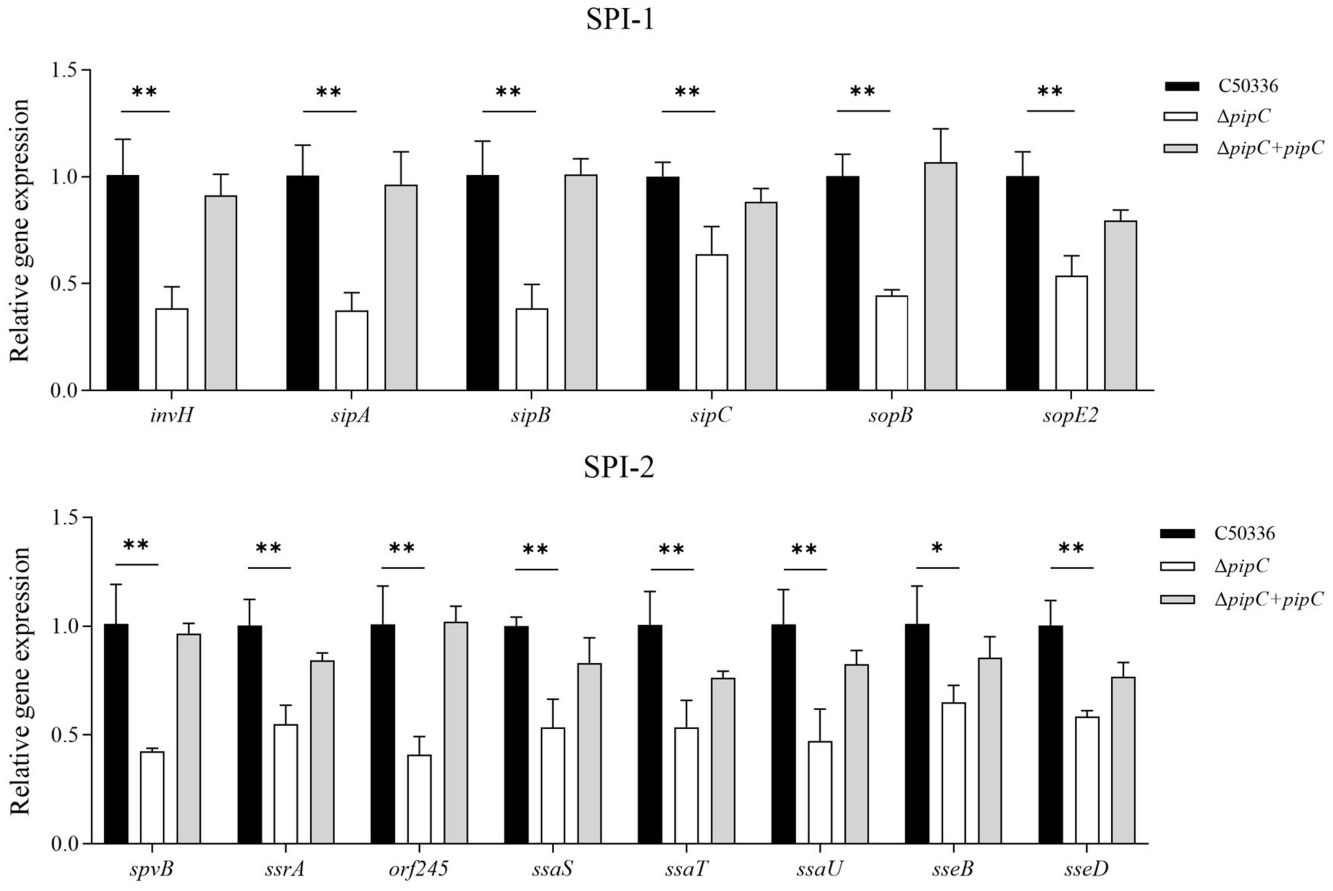
The expression level of virulence genes in C50336, Δ*pipC* and Δ*pipC* + *pipC* were detected by using qPCR, with 16*S rRNA* as the internal reference gene. Data are presented as the mean ± SD of three independent replicates. Statistical significance was determined as *p* < 0.05 (*) and *p* < 0.01 (**).

To investigate the potential mechanism underlying the attenuation of *SE* virulence due to the deletion of the *pipC* gene, qPCR was employed to assess the expression levels of virulence factors. The results are presented in Figure 5. Compared to C50336, the expression levels of SPI-1 associated genes (*invH*, *sipA*, *sipB*, *sipC*, *sopB*, and *sopE2*) and SPI-2 associated genes (*spvB*, *ssrA*, *orf245*, *ssaS*, *ssaT*, *ssaU*, *sseB*, and *sseD*) in Δ*pipC* were significantly decreased. These findings suggest that *pipC* may influence the virulence of *SE* by modulating the expression levels of multiple virulence genes.

### The deletion of *pipC* reduces the colonization of *SE* in organs

**Figure 6:**
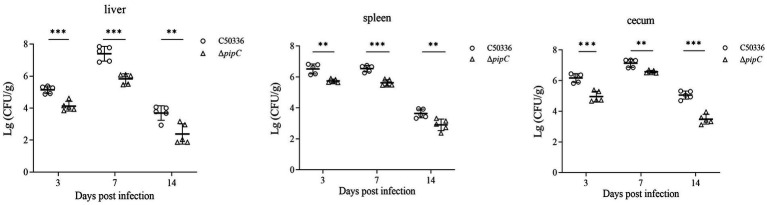
Bacterial colonization of *SE* in the liver, spleen, and cecum following challenge. Bacterial loads in these organs were quantified at the 3 dpc, 7 dpc, and 14 dpc, and results were expressed as log₁₀(CFU/g). Data represents the mean ± SD from five mice. Statistical significance is indicated as *p* < 0.01 (**) and *p* < 0.001 (***).

The results of bacteria colonization in the liver, spleen, and cecum are shown in Figure 6. All the liver, spleen and cecum samples from the blank control group were negative for *Salmonella* recovery. Bacteria could be isolated from the liver, spleen, and cecum at 3, 7, and 14 dpc. Compared to C50336, the counts of Δ*pipC* in the liver, spleen, and cecum were significantly lower at 3, 7, and 14 dpc, indicating that the colonization ability of Δ*pipC* in these organs was notably inferior to that of C50336. This suggests that the *pipC* gene can influence the colonization ability and virulence of *SE*.

### Δ*pipC* can induce immune responses

**Figure 7:**
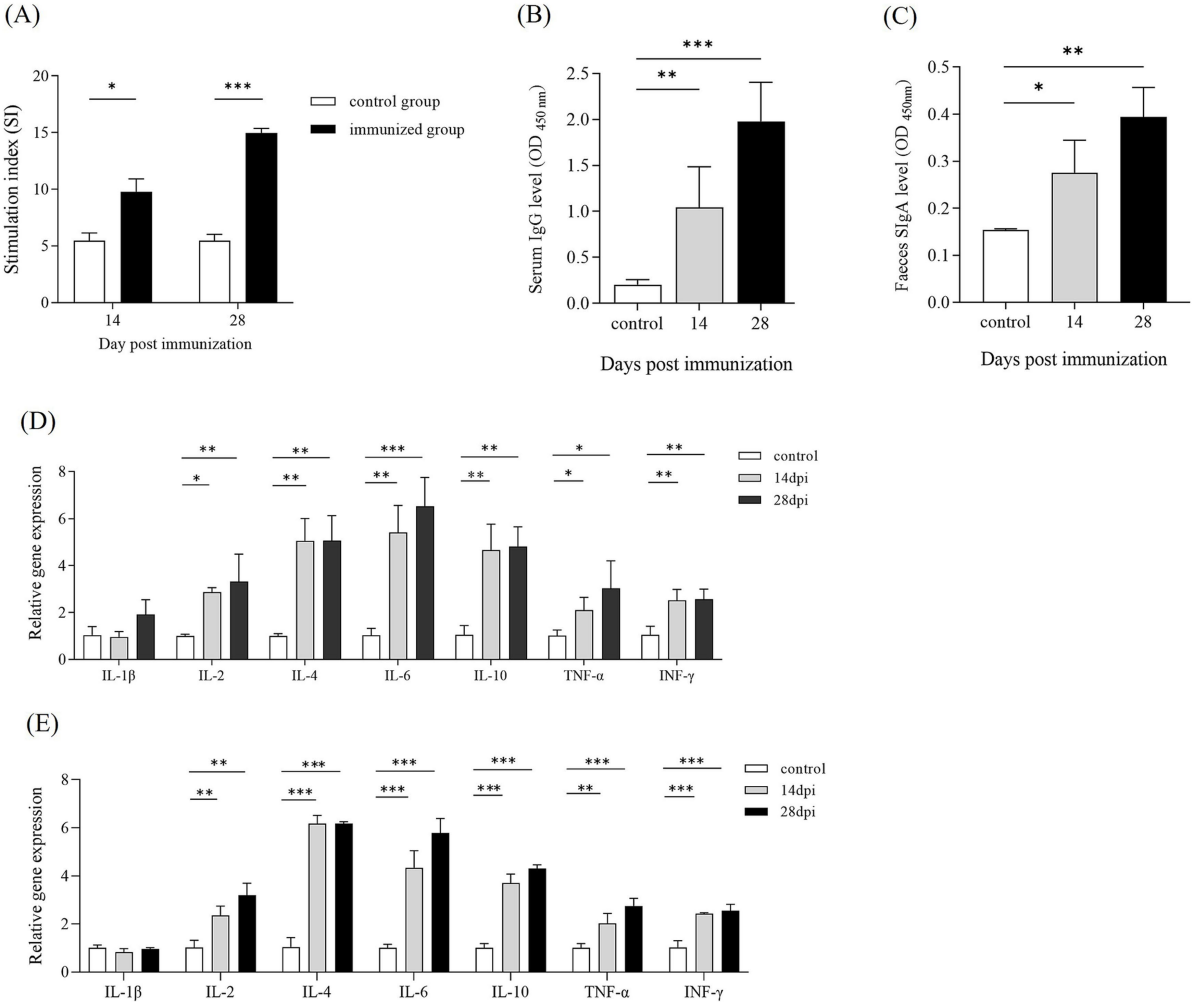
**(A)** The stimulation index (SI) of the splenic lymphocytes proliferation assay. Lymphocyte proliferation was measured with a MTT kit at 14 and 28 dpi. The SI was calculated using the following equation: SI = (OD_570_ of the antigen-stimulated cells)/(OD_570_ of the unstimulated cells). Data represent the mean ± SD from five mice. **(B,C)** Antibody levels in serum and feces of KM mice following immunization. Serum and fecal samples were collected from KM mice at 14 and 28 dpi. The levels of IgG in serum and IgA in feces were determined by ELISA. Data are presented as the mean ±SD from three independent replicates. **(D,E)** Cytokine expression levels in the spleen following immunization. Quantitative PCR analysis was performed to assess the mRNA expression levels of IL-1β, IL-2, IL-4, IL-6, IL-10, TNF-α, and IFN-γ at 14 and 28 dpi. The internal control gene were gapdh and β-actin, respectively. Data are presented as the mean ± SD of three independent replicates. Statistical significance was determined as *p* < 0.05 (*), *p* < 0.01 (**) and *p* < 0.001 (***).

To elucidate the specific immune responses to *SE* antigens following Δ*pipC* immunization, a splenic lymphocyte proliferation assay was performed using soluble antigens at 14 and 28 dpi. As shown in Figure 7A, the stimulation indices against the *SE* antigens for immunized group was 8.60 ± 0.34 at 14 dpi. Likewise, the stimulation indices against *SE* antigens for immunized group was 14.07 ± 0.51 at 28 dpi. The SI value of splenic lymphocytes in the immunized group was significantly higher than that in the control group, and the lymphocyte proliferation level further increased after enhanced immunization. These findings indicate that Δ*pipC* can induce strong specific immune responses.

To evaluate the humoral and mucosal immune responses in mice immunized with Δ*pipC*, the serum IgG and mucosal IgA responses against *SE* soluble antigens were measured by ELISA. At 14 dpi and 28 dpi, mice immunized with Δ*pipC* exhibited significantly enhanced serum IgG levels (Figure 7B) and secretory IgA (SIgA) levels (Figure 7C) compared to the control group, with further increases observed after booster immunization. These findings indicate that Δ*pipC* effectively induces robust specific humoral and mucosal immune responses, which are augmented with booster immunization.

We detected the expression of cytokines in spleen cells of immunized mice at 14 and 28 dpi. The results, shown in Figures 7D,E, revealed that at 14 dpi, the expression levels of IL-2, IL-4, IL-6, IL-10, TNF-α, and IFN-γ in the Δ*pipC* group were significantly higher than those in the control group. At 28 dpi, the expression levels of IL-2, IL-4, IL-6, IL-10, TNF-α, and IFN-γ in the Δ*pipC* group were notably higher than those in the control group. Additionally, the expression levels of IL-6 and TNF-α after the booster immunization were significantly higher than those after the primary immunization. The increased expression levels of cytokines highlight that Δ*pipC* can effectively induce a strong immune response in mice.

### Immune protection by the Δ*pipC* vaccination against virulent C50336 challenge

**Figure 8:**
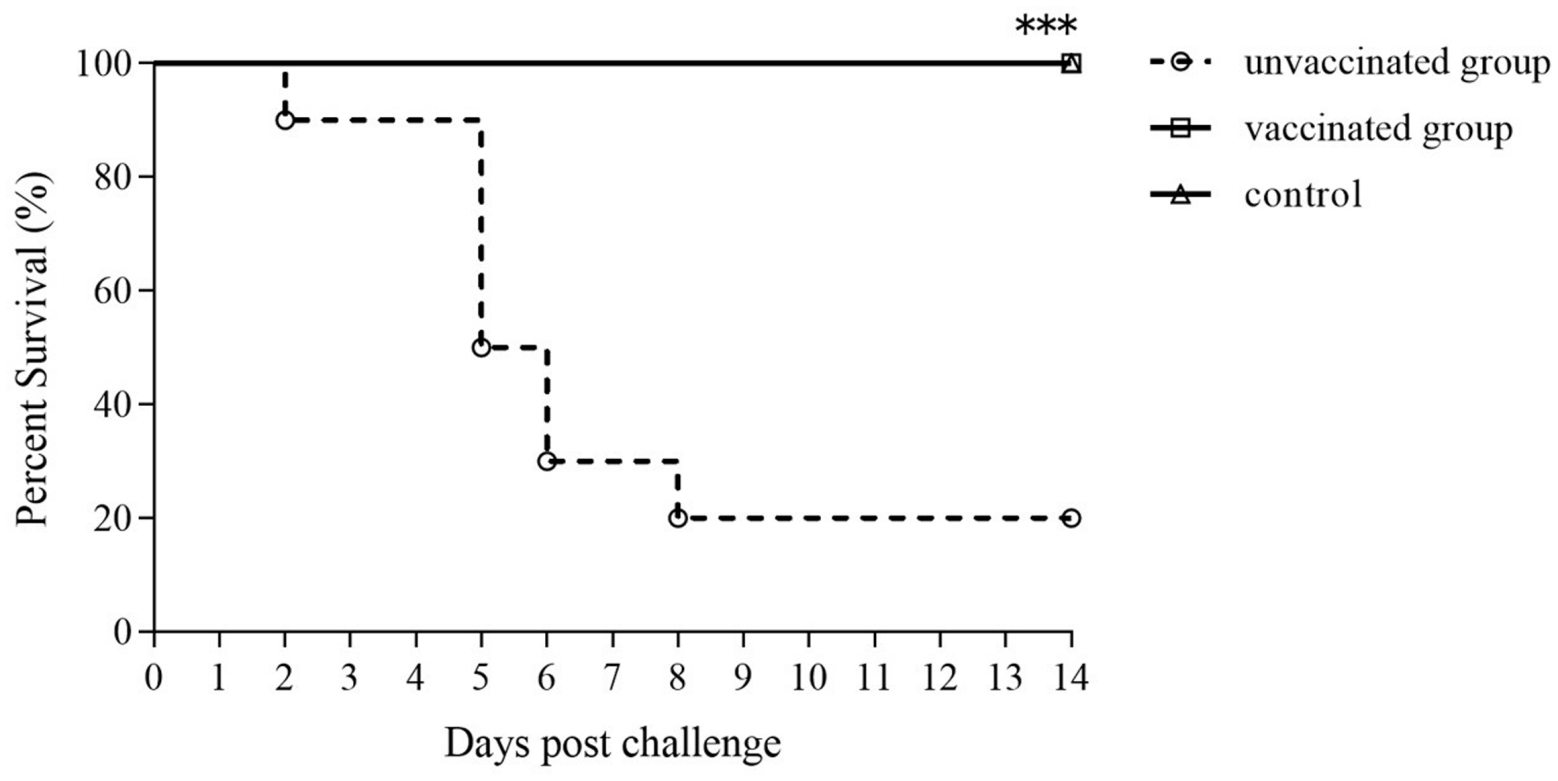
Protective efficacy of the Δ*pipC* following oral vaccination. KM mice were orally immunized with the Δ*pipC* and challenged with a lethal dose of C50336 at 28 dpi. Survival was monitored daily for 14 days after the challenge. The vaccinated group showed significantly improved survival compared to the unvaccinated group. Statistical significance was determined as *p* < 0.001 (***).

**Figure 9:**
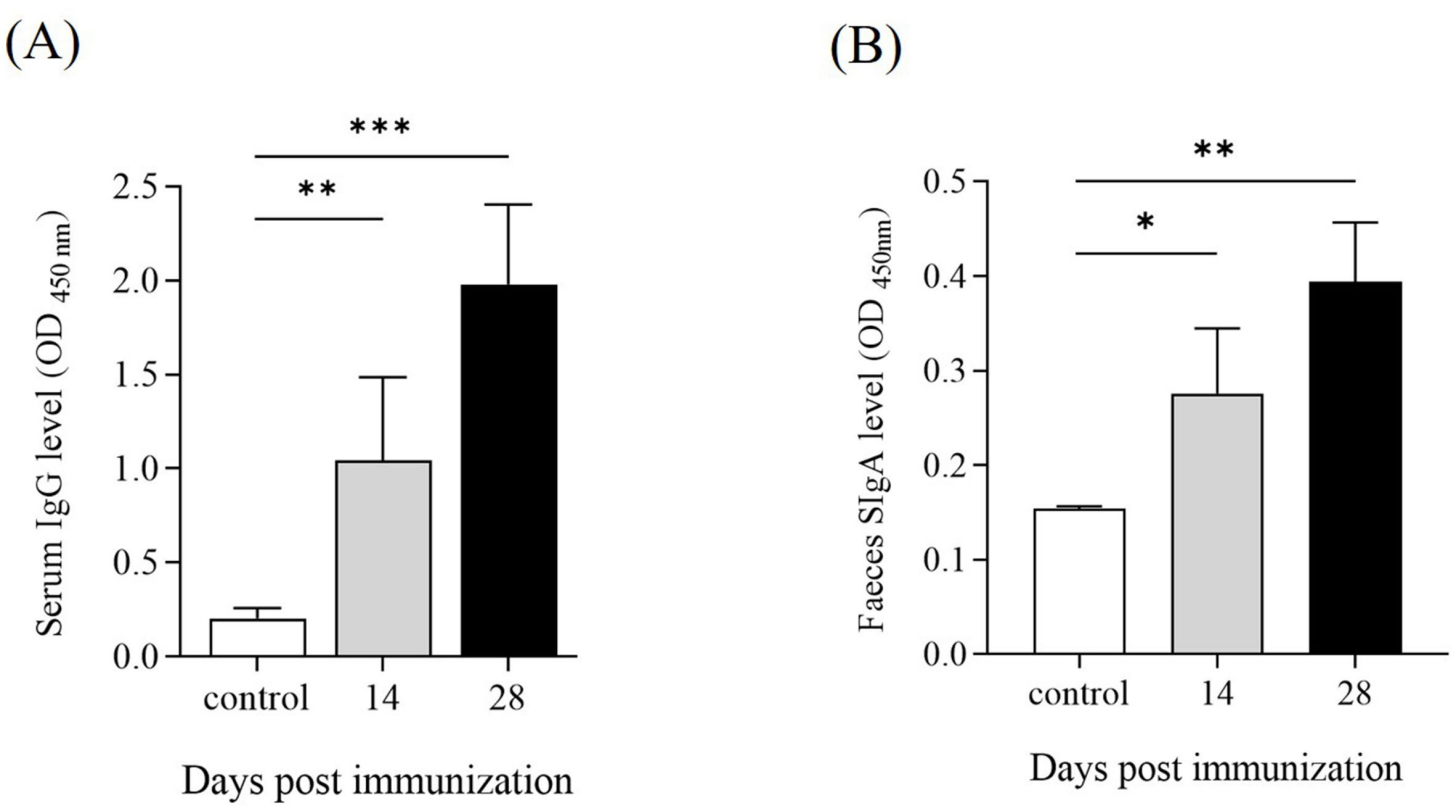
Antibody levels in serum and feces of KM mice following immunization. Serum and fecal samples were collected from KM mice at 14 and 28 days post-immunization (dpi). The levels of **(A)** IgG in serum and **(B)** IgA in feces were determined by ELISA. Data are presented as the mean ±SD from three independent replicates. Statistical significance was assessed as *p* < 0.05 (*), *p* < 0.01 (**) and *p* < 0.001 (***).

The survival percentages in the mice vaccinated orally with the Δ*pipC* followed by the challenge with the virulent *SE* are shown in Figure 8. The vaccinated group showed no mouse deaths and a 100% survival rate; among the 10 mice in the unvaccinated group, 8 mice died after the challenge, showing a survival rate of 20%. The clinical symptoms including anorexia, chills, diarrhea, emaciation, and depression in the vaccinated group were slight and temporary after challenged compared to the unvaccinated group. Therefore, immunization with 10^6^ CFU of Δ*pipC* provided full protection against *SE* challenge (see Figure 9).

## Discussion

*SE* is a major foodborne zoonotic pathogen that poses significant economic burdens on the livestock industry and presents a serious global public health threat. The continued emergence of antimicrobial resistance (AMR), particularly multidrug resistance (MDR), in *Salmonella* has become a growing challenge worldwide. Vaccination has been recognized as an effective targeted strategy to control antibiotic-resistant *Salmonella* infections. Studies have demonstrated that vaccination significantly contributes to reducing infections caused by resistant strains (Bian et al., 2024).

Over the years, a variety of vaccines-including inactivated and live attenuated vaccines-have been developed to combat salmonellosis. Inactivated vaccines are considered safe but generally induce weak immunogenicity, often requiring adjuvants and booster immunizations, and mainly stimulate humoral rather than cellular immunity (Pan et al., 2024). In contrast, live attenuated vaccines represent a major advancement in vaccine technology by providing efficient and long-lasting protection. These vaccines use weakened but viable pathogens that are capable of eliciting strong immune responses without causing disease. A key advantage of live attenuated vaccines lies in their ability to closely mimic natural infections and engage both humoral and cell-mediated immune responses, thereby establishing robust and sustained protection (Nazir et al., 2025).

In recent years, the use of targeted gene deletion to attenuate virulence in *Salmonella* has become an increasingly common strategy in vaccine development (Hewawaduge et al., 2023). Given the association of *pipC* with host cell invasion and intracellular survival in macrophages, we constructed a *pipC*-deleted *SE* mutant using homologous recombination. Compared to the wild-type strain C50336, the Δ*pipC* mutant exhibited significantly reduced virulence. Immunization with the Δ*pipC* strain induced strong humoral and cellular immune responses and conferred full protection against subsequent *SE* challenge in mice.

Identification of virulence genes is essential for the rational design of live attenuated vaccines. During infection, *SE* faces various stress conditions--such as acid, alkali, and oxidative stress—both in the gastrointestinal tract and within *Salmonella*-containing vacuoles (SCVs) of epithelial and macrophage cells. Genes involved in stress responses are often linked to virulence (Pasqua et al., 2022). Our study demonstrated that deletion of *pipC* had no significant effect on the growth characteristics of *SE* under normal conditions, indicating that this gene is not essential for basic bacterial proliferation. However, the *pipC* mutant strain exhibited markedly reduced survival under acidic, alkaline, and oxidative stress conditions, suggesting that *pipC* is involved in the bacterium’s ability to withstand environmental stress. These findings underscore the important role of *pipC* in stress resistance, which may, in turn, contribute to the overall virulence and persistence of *SE* in hostile environments.

Upon entering the small intestine, *Salmonella* first adheres to epithelial cells and invades host tissues via M cells located over Peyer’s patches. Although macrophages within lymphoid tissues phagocytose *Salmonella*, the bacterium can evade destruction by interfering with phagosome-lysosome fusion, allowing it to survive and replicate intracellularly. Hence, adhesion to epithelial cells and intracellular survival in macrophages are crucial virulence determinants (Eakley et al., 2011). In this study, we found that the Δ*pipC* strain exhibited significantly reduced invasion of epithelial cells, consistent with findings by Shah et al. (2011). Additionally, intracellular proliferation was markedly impaired. A mouse intraperitoneal infection model showed that the LD₅₀ of the Δ*pipC* strain increased 47-fold compared to the wild-type strain, indicating substantial attenuation. The SPI-1 and SPI-2 pathogenicity islands encode the Type III Secretion System (T3SS), which delivers effector proteins into host cells to facilitate cytoskeletal rearrangement and invasion. Gene expression analysis further revealed that the Δ*pipC* strain had significantly downregulated expression of several virulence-associated genes. SPI-1 genes (*invH*, *sipA*, *sipB*, *sipC*, *sopB*, and *sopE2*) and SPI-2 genes (*spvB*, *ssrA*, *orf245*, *ssaS*, *ssaT*, *ssaU*, *sseB*, and *sseD*) were all suppressed. The downregulation of these genes in the Δ*pipC* strain implies impaired T3SS function and effector protein expression, ultimately leading to reduced invasion and virulence.

Live attenuated *Salmonella* vaccines must balance attenuation, immunogenicity, and protective efficacy. An ideal vaccine strain should be non-toxic or minimally toxic. In this study, mice infected with the Δ*pipC* strain had significantly higher LD₅₀ values and reduced colonization in the intestine, spleen, and liver compared to wild-type infections, confirming the attenuation of the mutant strain.

Upon entry, *SE* activates innate immunity, recruiting macrophages and monocytes, which secrete pro-inflammatory cytokines and promote inflammation. In parallel, B cells differentiate into effector cells, producing anti-inflammatory cytokines such as IL-10, which suppress inflammation and stimulate antibody production. Cytokines such as IFN-γ enhance macrophage activation and help regulate immune responses (Jan et al., 2022; Kung et al., 2022). In our study, mice immunized with the Δ*pipC* strain showed significantly higher splenic lymphocyte proliferation indices and serum antibody levels at both 14 dpi and 28 dpi compared to controls. Cytokine analysis revealed significantly increased expression of IL-2, IL-4, IL-6, IL-10, TNF-α, and IFN-γ, indicating robust activation of both humoral and cellular immune pathways. The Δ*pipC* strain provided complete protection (100%) against secondary wild-type challenge when administered orally at a dose of 1 × 10^6^ CFU. It is important to note that we used only a single antigen concentration in our lymphocyte proliferation experiments, limiting the understanding of antigen sensitivity and cellular response thresholds. Assessment of lymphocyte response at multiple antigen concentrations should be considered in future further studies.

One of the main advantages of live attenuated vaccines over inactivated ones is their ability to stimulate both humoral and cellular immunity. Systemic dissemination of *Salmonella* promotes antigen presentation and induces specific immune responses that prevent secondary infections. Previous studies have validated the potential of gene-engineered live attenuated vaccines against *Salmonella*. Zhao et al. (2024) showed that deletion of the *pal* gene attenuated *SE* virulence, and oral immunization with 1 × 10^8^ CFU of Δ*pal* conferred 100% protection against a 5 × 10^6^ CFU wild-type challenge. Zhou et al. (2023) demonstrated that deletion of *rfbG* increased the LD₅₀ by 56-fold. Immunizing chickens with 5 × 10^7^ or 5 × 10^6^ CFU of Δ*rfbG* achieved 100% survival after wild-type challenge, and significant antibody responses were observed, confirming its vaccine potential. Similarly, Pan et al. (2020) reported that the LD₅₀ of *Salmonella Paratyphi A* Δ*sptP* was 1.43 × 10^4^-fold higher than the wild-type, and oral immunization with 2 × 10^5^ CFU conferred complete protection against challenge with 1 × 10^3^ CFU.

In summary, our present work demonstrates that lack of *pipC* affects *SE* pathogenicity by decreasing its virulence both *in vitro* and *in vivo*. Vaccination of mice with Δ*pipC* conferred development of acquired immunity and efficacious protection against experimental systemic infection. The *pipC* mutant possesses the safety and efficacy required for use as a live attenuated vaccine. Given the potential for field applications, a more comprehensive long-term assessment of safety indicators such as fecal-shedding duration, risk of virulence re-escalation, and potential for environmental transmission is needed (Pan et al., 2020). Although the Δ*pipC* strain exhibited attenuated virulence and genetic stability under laboratory conditions, its environmental safety and stability should still be verified by field experiments before practical application, and the applicability of target animals and the safe window of vaccine dose should be systematically evaluated.

## Data Availability

The original contributions presented in the study are included in the article/supplementary material, further inquiries can be directed to the corresponding author.

## Supplementary material

SUPPLEMENTARY FIGURE 1 **(A)** Schematic representation of a gene knockout strategy adopted from Ranallo et al. (2006). **(a)** Linear DNA substrates containing chloramphenicol cassettes are generated using PCR primers with 59 bp homology (A and B) to the gene of interest (*pipC*). Priming from pKD3 produces linear DNA substrates. **(b)** These substrates introduced into bacteria made transiently hyper-recombinogenic using Gam, Beta, Exo expressed from pKD46. **(c)** The chloramphenicol cassette is eliminated via plasmid-based expression of a yeast derived recombinase (FLP) leaving behind an ~80 bp “scar” consisting of a single FRT site. **(B)** Schematic representation of *pipC*-complemented strain construction of *SE*.
