# Supplementary figures and images for: PipC affects the virulence of Salmonella enterica serovar Enteritidis and its deletion strain provides effective immune protection in mice
Source: Front Microbiol. 2025 Jun 24;16:1631008. doi: 10.3389/fmicb.2025.1631008 (PMC12234537; doi:10.3389/fmicb.2025.1631008)

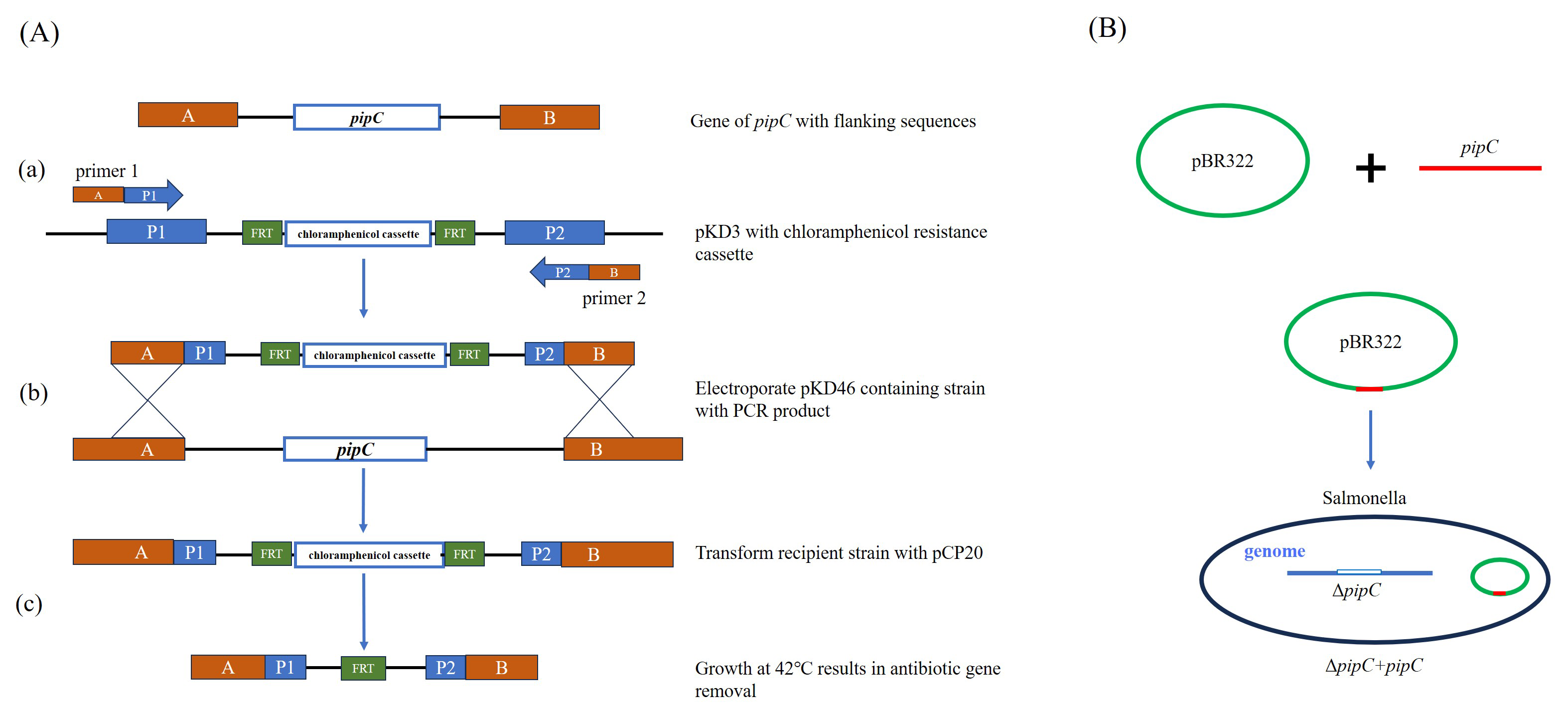

Supplement: SUPPLEMENTARY FIGURE 1 — (A) Schematic representation of a gene knockout strategy adopted from Ranallo et al. (2006). (a) Linear DNA substrates containing chloramphenicol cassettes are generated using PCR primers with 59 bp homology (A and B) to the gene of interest (pipC). Priming from pKD3 produces linear DNA substrates. (b) These substrates introduced into bacteria made transiently hyper-recombinogenic using Gam, Beta, Exo expressed from pKD46. (c) The chloramphenicol cassette is eliminated via plasmid-based expression of a yeast derived recombinase (FLP) leaving behind an ~80 bp “scar” consisting of a single FRT site. (B) Schematic representation of pipC-complemented strain construction of SE. [file Image_1.tif]
